# Supplementary material for: Unsupervised Machine Learning Reveals Temporal Components of Gene Expression in HeLa Cells Following Release from Cell Cycle Arrest
Source: Int J Mol Sci. 2025 Sep 28;26(19):9491. doi: 10.3390/ijms26199491 (PMC12524476; doi:10.3390/ijms26199491)
Supplement: Supplementary file 1 [file ijms-26-09491-s001.zip › ijms-3722788-supplementary.pdf]

Supplementary text and figures for

Unsupervised machine learning reveals temporal components of gene expression in HeLa cells following release from cell cycle arrest

Tom Maimon[1], Yaron Trink[1], Jacob Goldberger[1], and Tomer Kalisky [1,2]

[1]Faculty of Engineering and Bar-Ilan Institute of Nanotechnology and Advanced Materials (BINA), Bar-Ilan University, Ramat Gan, Israel 5290002

[2]Correspondence to:

Tomer Kalisky

Faculty of engineering, Bar-Ilan University, Ramat Gan, Israel 52900

Office: +972-3-738-4656

Email: tomer.kalisky@biu.ac.il

FIGURES

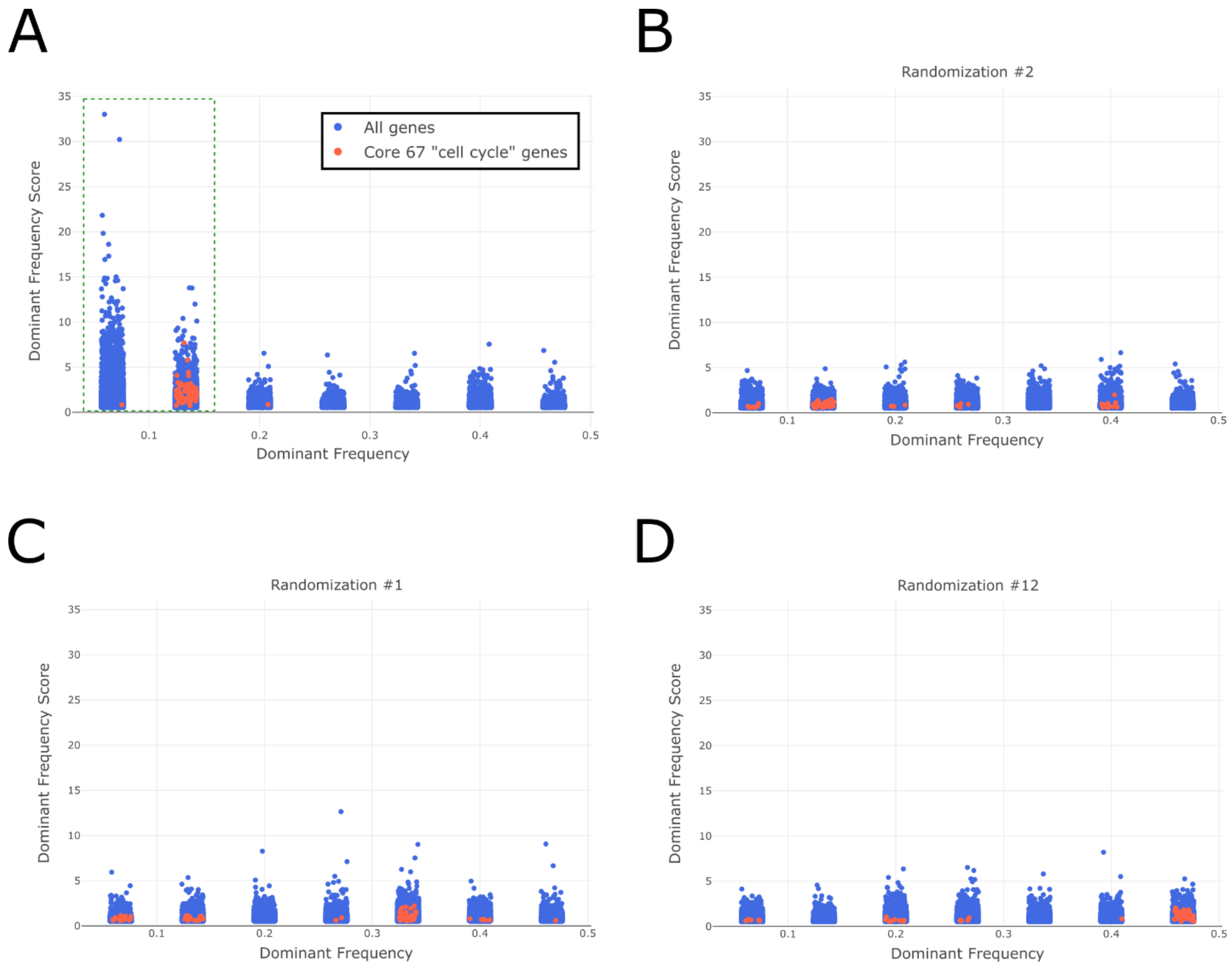

Figure S1: Fourier analysis identifies sets of genes with potentially transient and oscillatory behaviors over time.

(A) A periodogram was calculated for each gene, and its dominant frequency vs. dominant frequency score were plotted (see Methods). It can be seen that genes (=dots) within the first and second dominant frequencies have scores that are higher than genes within other dominant frequencies. Likewise, the genes within the 3rd dominant frequency and onwards have scores that are similar to those derived from randomized datasets, which were generated by randomly shuffling the order of counts for each gene (B-D). This indicates that the genes within the first and second dominant frequencies contain most of the periodic information in our dataset.

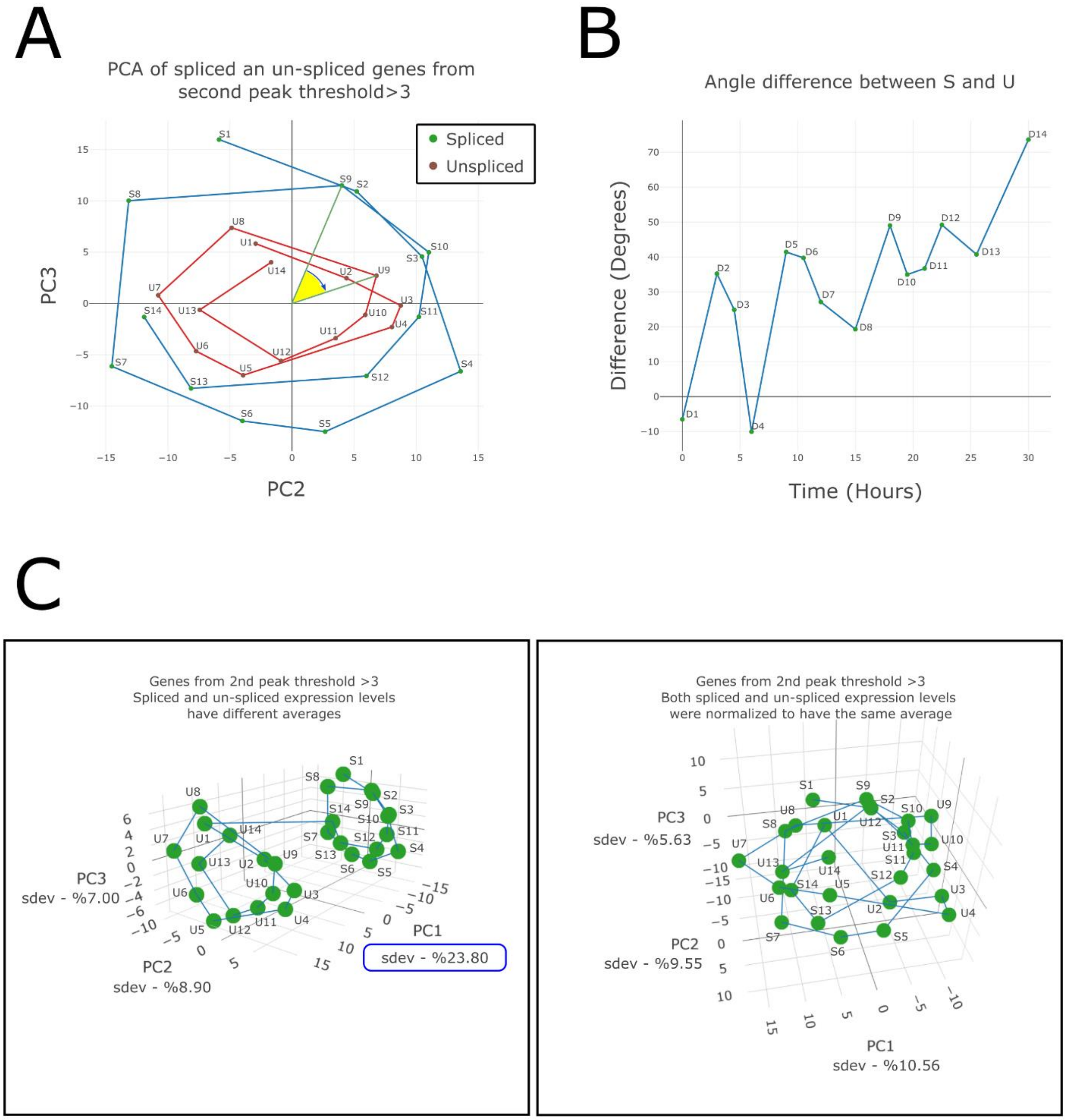

Figure S2: RNA velocity analysis of periodically expressed genes reveals a time lag between spliced and un-spliced mRNA.

(A) After performing Fourier analysis, selecting only genes from the second dominant frequency with scores > 3, and performing PCA, the samples form a circular trajectory in latent space that represents almost two complete cell cycles. Further separation into spliced (S1, S2, S3, …) and un-spliced (U1, U2, U3, …) mRNA expression profiles results in two circular patterns in latent space with a rotation angle difference between them (note: the unspliced circle was artificially

made smaller in order to assist visibility) (B) This difference in rotation angle appears to start from zero, increase, and then stabilize over time at some constant value. However, longer and more frequent measurements will be needed to validate this observation. (C) Note that the two circles representing the spliced and un-spliced data points are oriented in parallel to the PC2 vs. PC3 plane and are widely separated along the PC1 axis as a result the difference in average expression between the spliced and un-spliced mRNA in each gene (left panel). Normalizing the spliced and un-spliced expression profiles to have the same average expression in each gene results in both circles collapsing on the same plane in latent space, that is, the PC1 vs. PC2 plane, with negligible separation along the PC3 axis (right panel).

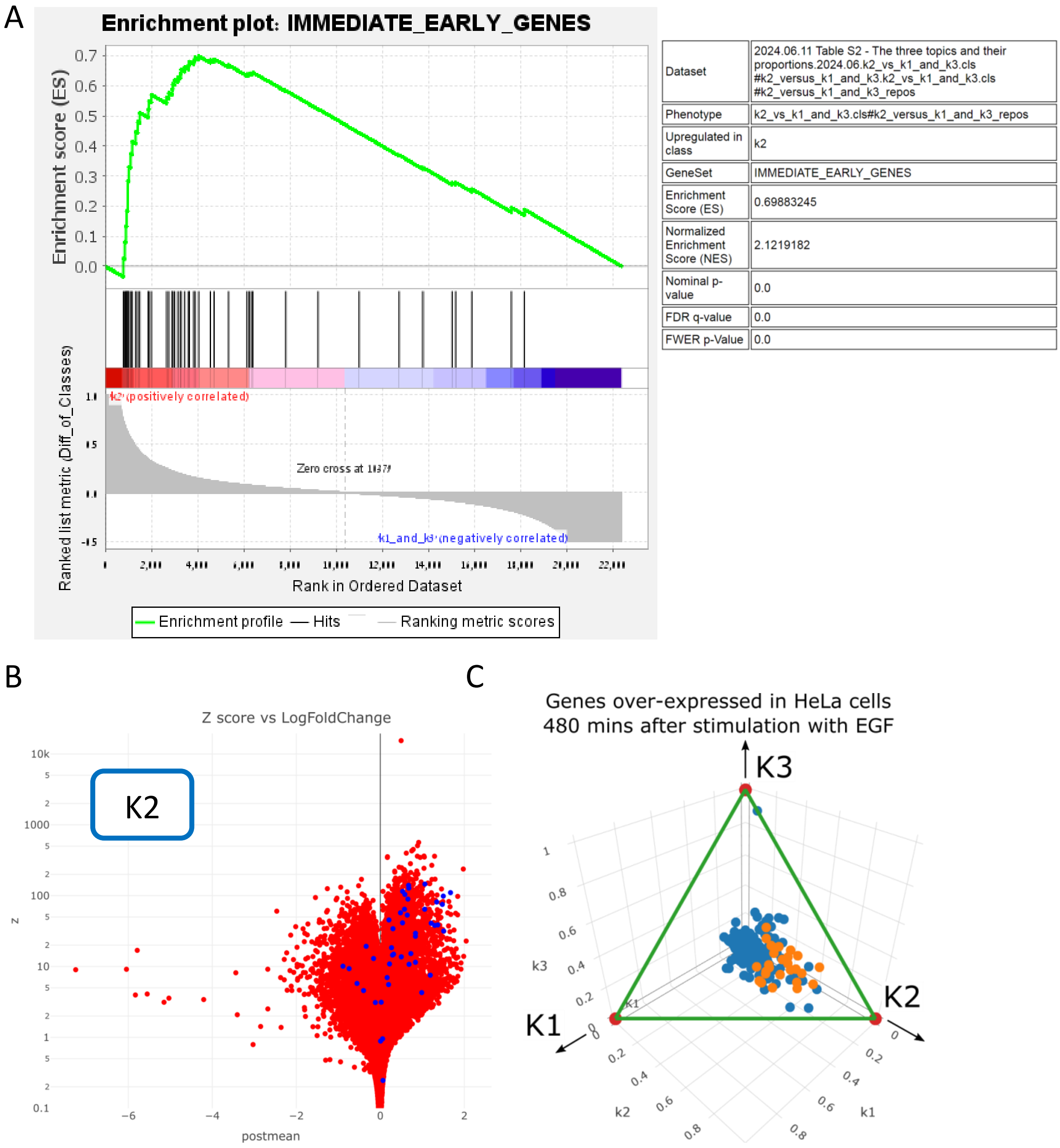

Dataset	2024.06.11 Table S2 - The three topics and their proportions.2024.06.k2_vs_k1_and_k3.cls #k2_versus_k1_and_k3.k2_vs_k1_and_k3.cls #k2_versus_k1_and_k3_repos
Phenotype	k2_vs_k1_and_k3.cls#k2_versus_k1_and_k3_repos
Upregulated in class	k2
GeneSet	IMMEDIATE_EARLY_GENES
Enrichment Score (ES)	0.69883245
Normalized Enrichment Score (NES)	2.1219182
Nominal p-value	0.0
FDR q-value	0.0
FWER p-Value	0.0

Figure S3: Immediate-early response genes are associated with topic k2.

(A) A GSEA enrichment plot shows that the immediate-early response genes are significantly over-expressed in topic k2 vs. topics k1 and k3 (Enrichment Score = 0.69, Normalized

Enrichment Score = 2.12, p_value < 10^{-3}). (B) A volcano plot shows that a large proportion of immediate-early response genes are over-expressed in topic k2. Each data point represents a gene, where the immediate-early response genes are colored in blue. (C) A posterior probability plot demonstrates similar enrichment for a set of genes that showed transient over-expression following stimulation with EGF (epidermal growth factor), a component of calf serum that also stimulates expression of immediate-early response genes [1].

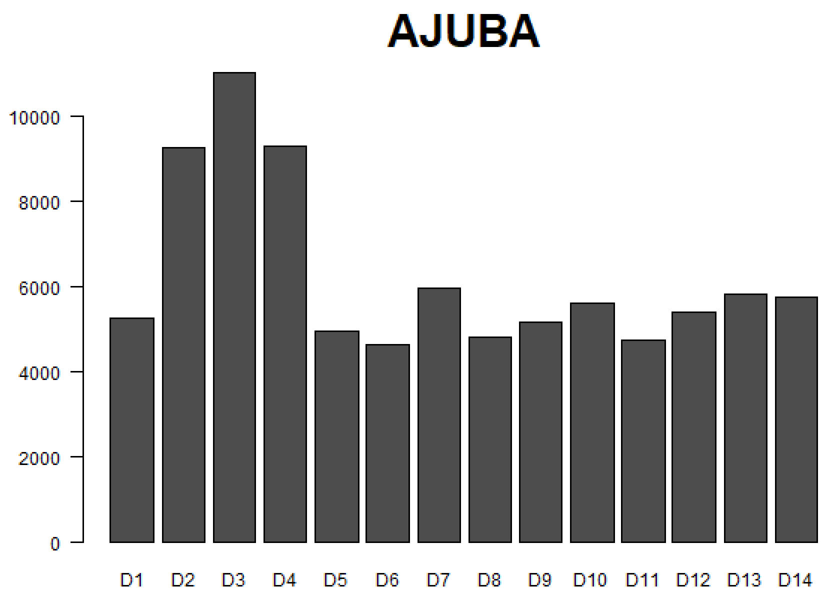

Figure S4: The gene *AJUBA* is transiently over-expressed following release from cell cycle arrest and is associated with topic k2.

The gene *AJUBA* was previously found to be over-expressed in cervical cancer [2] and its depletion was found to cause S-phase delay in cell lines [3].

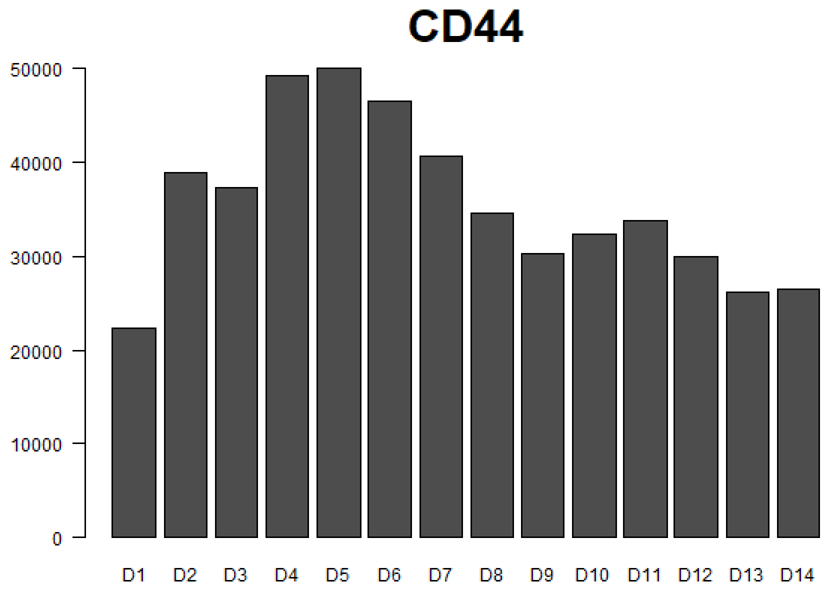

Figure S5: The gene *CD44* is transiently over-expressed following release from cell cycle arrest and is associated with topic k2.

The expression of the gene *CD44* was previously found to increase with progression from normal cervical epithelium to high-grade squamous intraepithelial lesions, and further with progression to squamous cell carcinoma of the cervix [4].

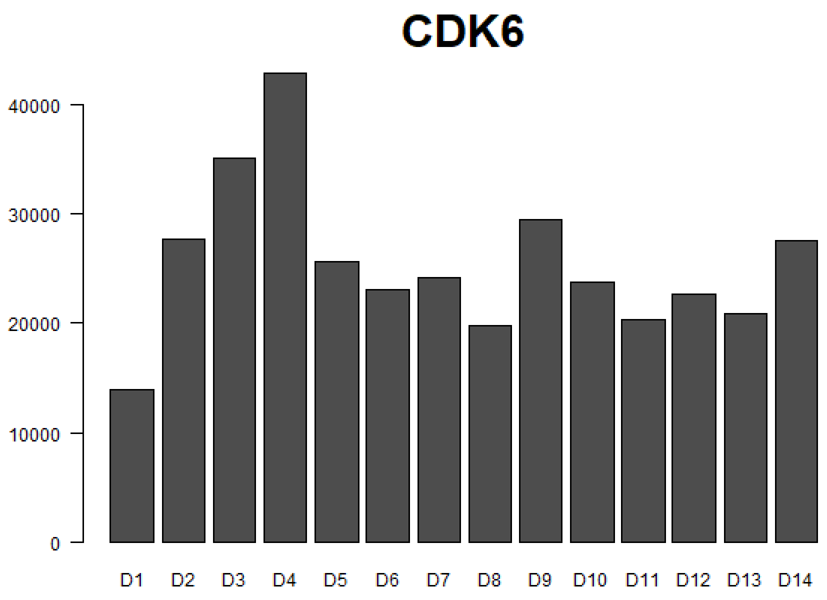

Figure S6: The gene *CDK6* is transiently over-expressed following release from cell cycle arrest and is associated with topic k2.

The gene *CDK6* is thought to be associated with the onset of cell cycle progression [5]. *CDK6* was previously found to be significantly over-expressed in cervical tumors, relative to both normal cervical epithelium and cervical intraepithelial neoplasia (CIN) [6].

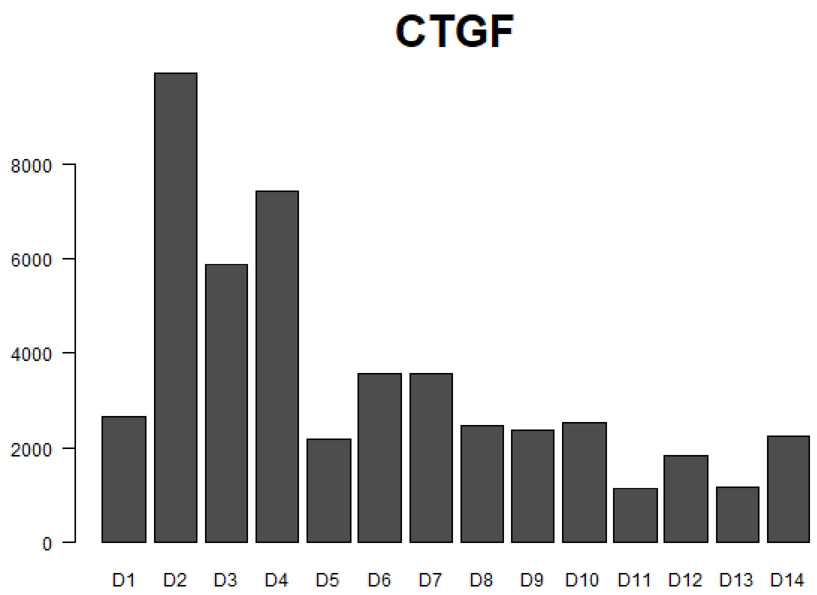

Figure S7: The gene *CTGF* (*CCN2*) is transiently over-expressed following release from cell cycle arrest and is associated with topic k2.

The gene *CTGF* (*CCN2*) is an immediate-early response gene [7]. This gene was previously found to be dysregulated in cervical cancer, showing downregulation in cancer compared to normal tissues [8], and upregulation in late stage cancer compared to early stage cancer [9].

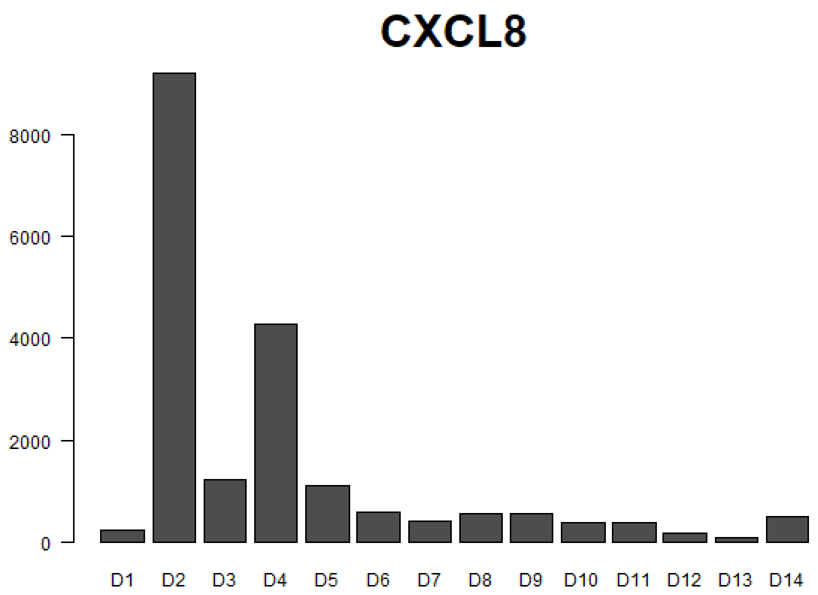

Figure S8: The gene *CXCL8* is transiently over-expressed following release from cell cycle arrest and is associated with topic k2.

The gene *CXCL8* was previously found to be overexpressed in cervical cancer biopsies relative to normal tissues [10], and higher expression levels of *CXCL8* were found to be related to a worse prognostic survival.

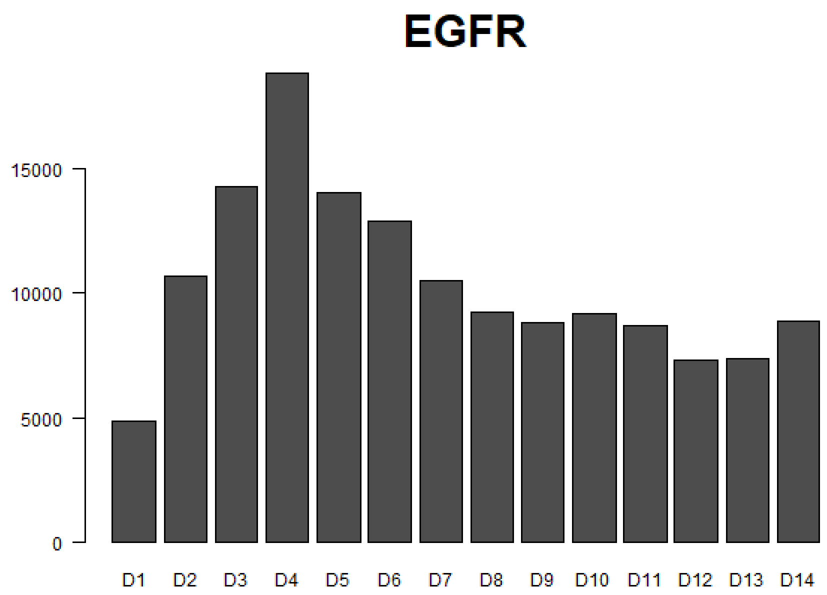

Figure S9: The gene *EGFR* is transiently over-expressed following release from cell cycle arrest and is associated with topic k2.

The gene *EGFR*, a receptor tyrosine kinase that converts extracellular cues into cellular responses such as cell proliferation, was previously found to be significantly over-expressed in patients with invasive cervical cancer, in both the primary tumors and the lymph node metastases [11,12].

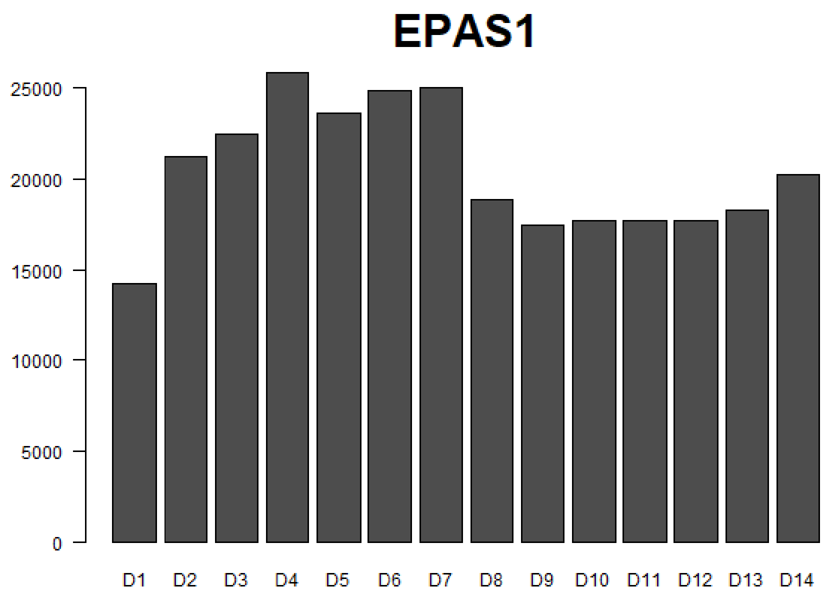

Figure S10: The gene *EPAS1* (*HIF2A*) is transiently over-expressed following release from cell cycle arrest and is associated with topic k2.

The gene *EPAS1* (*HIF2A*), a transcription factor that regulates genes involved in response to low oxygen concentration, was previously found to be over-expressed in Cervical Squamous Cell Carcinoma with respect to normal cervical tissues [13].

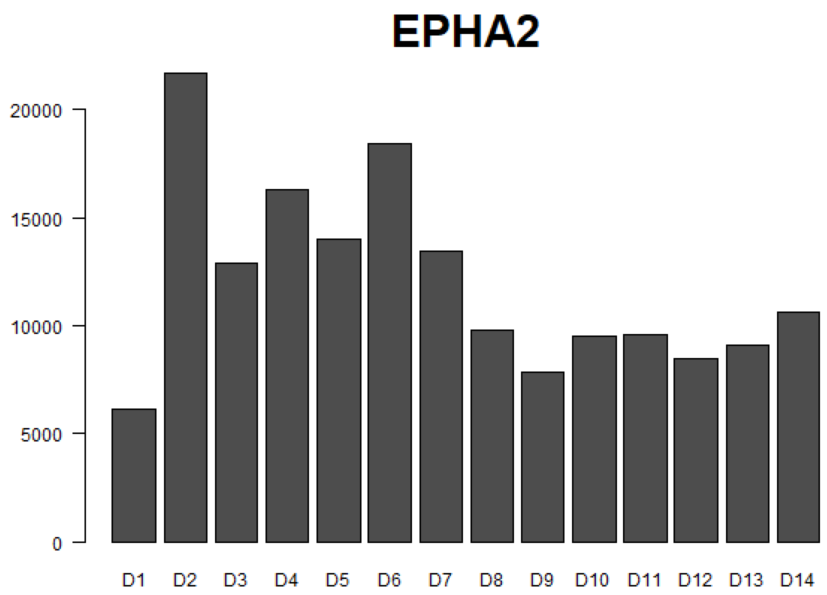

Figure S11: The gene *EPHA2* is transiently over-expressed following release from cell cycle arrest and is associated with topic k2.

The gene *EPHA2* was previously found to be highly expressed in cervical cancer relative to both normal cervical epithelium and cervical intraepithelial neoplasia (CIN) [6]. Elevated levels of *EPHA2* were also observed in advanced-stage tumors and tumors with lymph node metastasis.

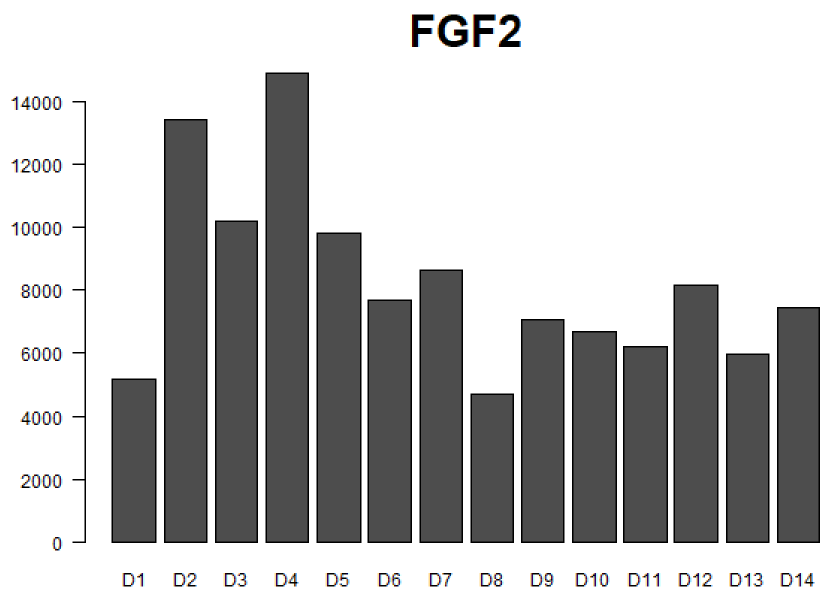

Figure S12: The gene *FGF2* is transiently over-expressed following release from cell cycle arrest and is associated with topic k2.

The gene *FGF2* (also known as basic fibroblast growth factor, or *BFGF*) was previously found to be highly expressed in advanced-stage cervical cancer, regardless of histological type [14].

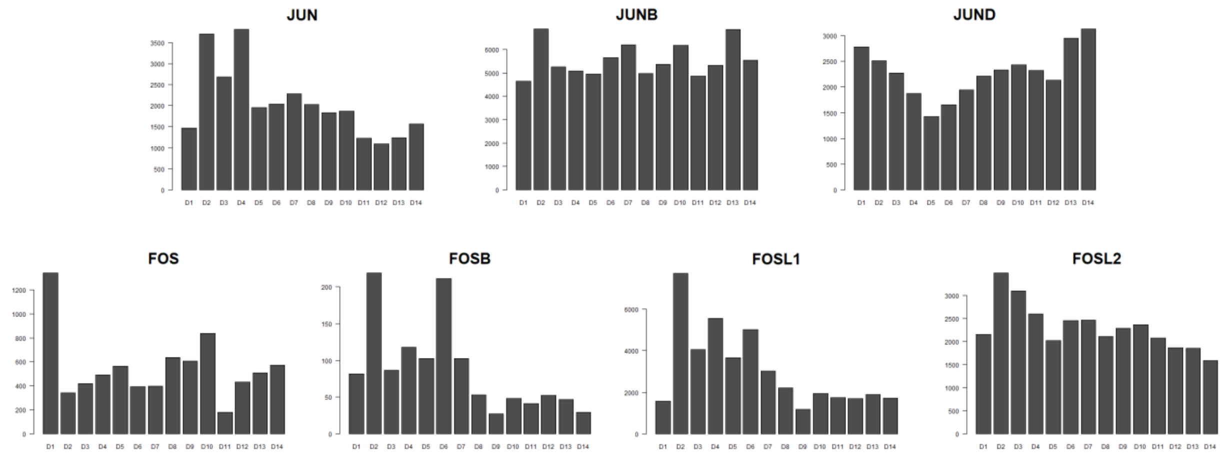

Figure S13: The genes *JUN* (C-Jun) and *FOSL1* (*FRA-1*) are transiently over-expressed following release from cell cycle arrest and are associated with topic k2.

The proteins encoded by the genes *JUN* (c-Jun), *JUNB*, *JUND*, *FOS* (c-Fos), *FOSB*, *FOSL1* (*FRA-1*), and *FOSL2* (*Fra-2*) can heterodimerize to form the AP-1 complex, a transcription factor known to be involved in cell proliferation and cancer progression [15–17]. Likewise, the genes *JUN*, *JUNB*, *FOS*, and *FOSB* were previously classified as immediate-early response genes [7]. It was previously found that downregulation of *JUN* inhibits the proliferation and invasion potential of HeLa cells [18]. Note, however, that the association of *FOSL1* to cervical cancer progression not straightforward. For example, *FOSL1* was found to gradually decrease during progression of normal tissue to early precancerous lesions and then to invasive cervical tumors [19]. Moreover, the high levels of *FOSL1* expression in the early precancerous cervical lesions were found to be associated with low overall AP-1 binding activity.

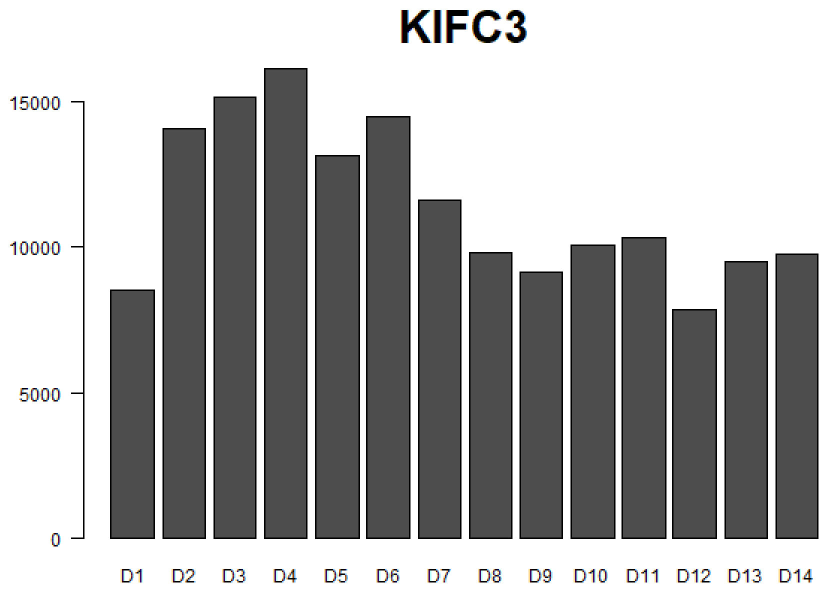

Figure S14: The gene *KIFC3* is transiently over-expressed following release from cell cycle arrest and is associated with topic k2.

The gene *KIFC3* was previously found to be associated with cell proliferation, migration, and invasion in colorectal cancer [20].

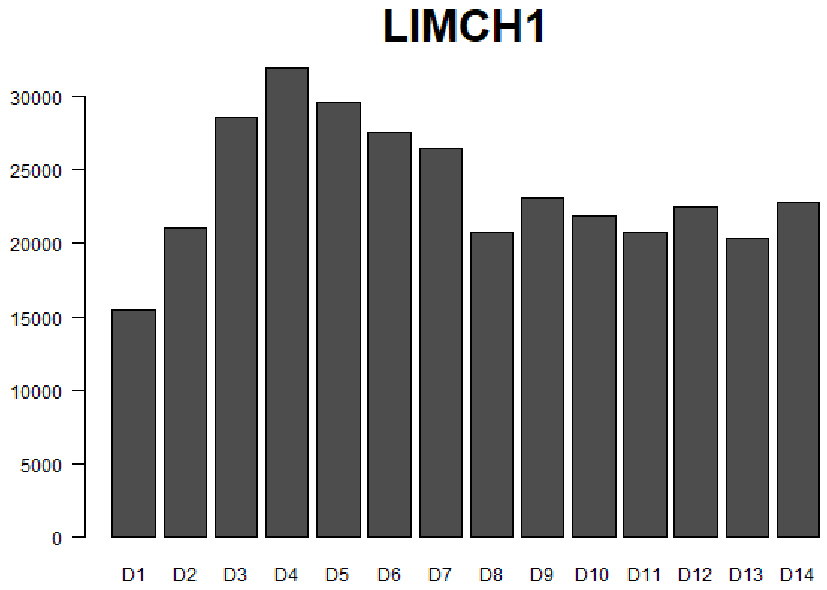

Figure S15: The gene *LIMCH1* is transiently over-expressed following release from cell cycle arrest and is associated with topic k2.

High expression levels of the gene *LIMCH1* were previously found to predict poor outcome in cervical cancer patients [21].

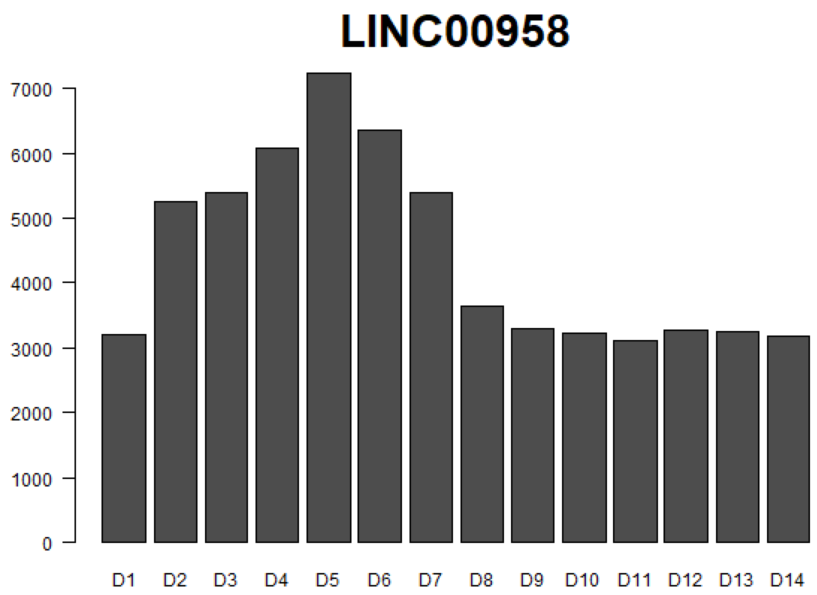

Figure S16: The long noncoding RNA *LINC00958* is transiently over-expressed following release from cell cycle arrest and is associated with topic k2.

The long noncoding RNA *LINC00958* was previously found to be overexpressed in cervical cancer and to be associated with cervical cancer cell proliferation and metastasis [22].

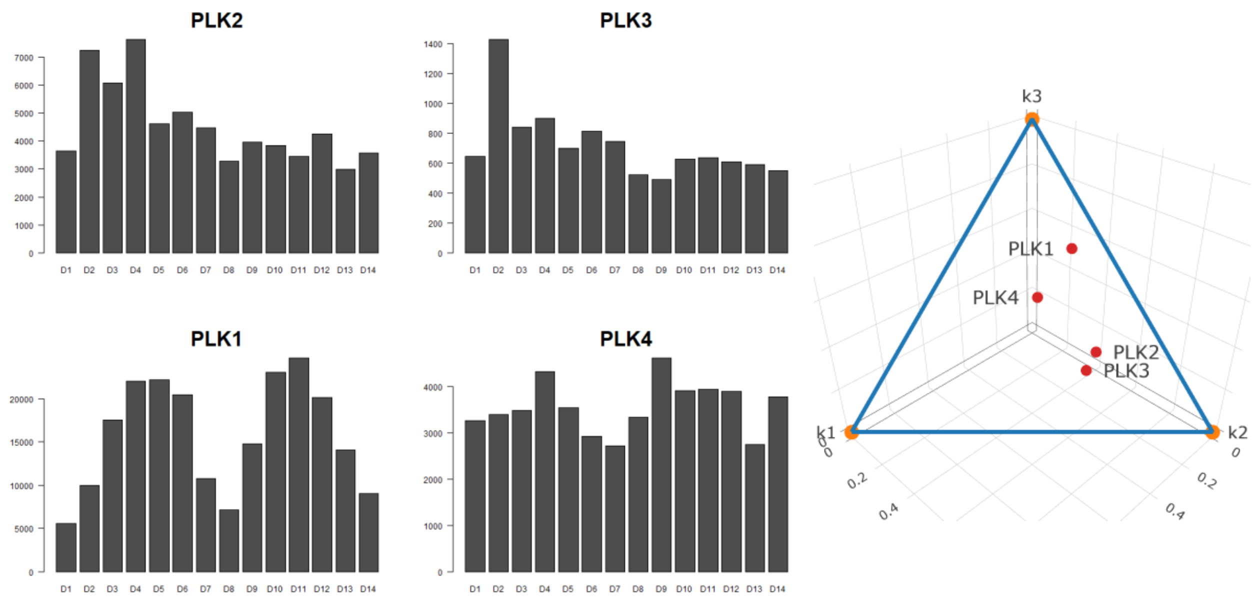

Figure S17: The genes *PLK2* and *PLK3* are transiently over-expressed following release from cell cycle arrest and are associated with topic k2.

The polo-like kinases *PLK1*, *PLK2*, *PLK3*, and *PLK4*, are associated with cell cycle regulation and progression [23]. *PLK1*, which is expressed in highly proliferating tissues, is known to perform multiple functions during the cell cycle [24]. On the other hand, *PLK2* and *PLK3*, which have a more broad tissue distribution, are considered immediate-early response genes [1]. In the dataset that we studied we observed that *PLK2* and *PLK3* are transiently over-expressed following release from cell cycle arrest, while *PLK1* (and *PLK4* to some extent) are modulated according to the cell cycle phase. The diagram of posterior probabilities (right) illustrates the association of *PLK2* and *PLK3* with topic k2, which represents transient expression following release from cell cycle arrest, and of *PLK1* with topic k3, linked to the G2-M phases of the cell cycle.

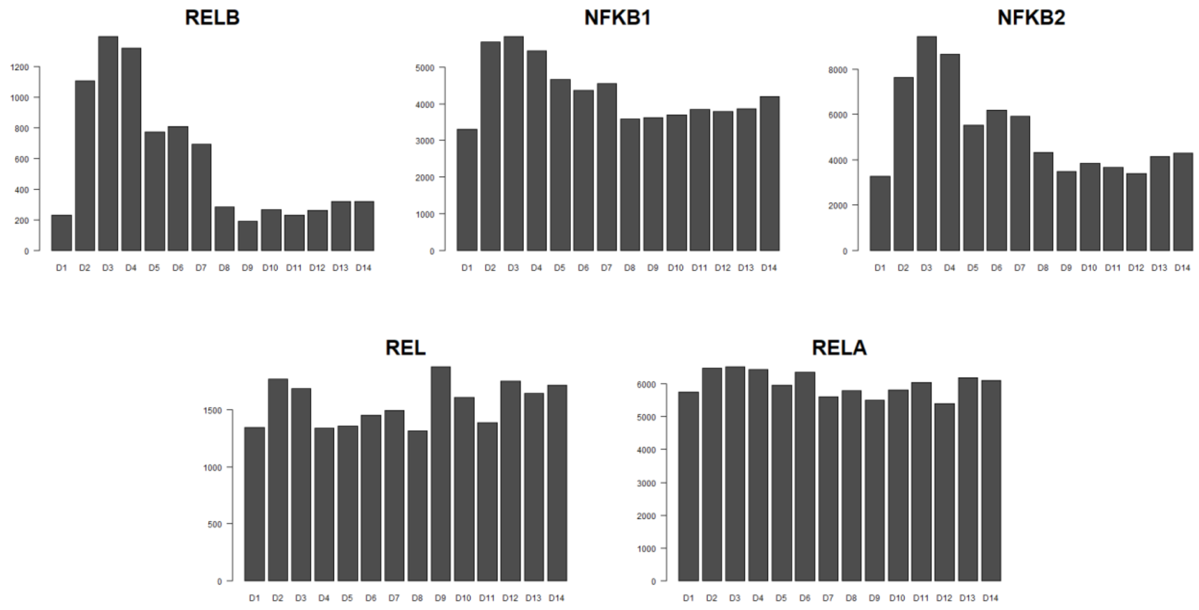

Figure S18: The genes *RELB*, *NFKB1*, and *NFKB2* are transiently over-expressed following release from cell cycle arrest and are associated with topic k2.

The genes *REL* (c-Rel), *RELA* (p65), *RELB*, *NFKB1* (p105/p50), and *NFKB2* (p100/p52), are members of the NF-kB family of transcription factors that are known to form various homo- or heterodimers. Upon activation these transcription factors can translocate to the nucleus, bind to DNA cis-regulatory elements at enhancers and promoters, and induce target genes involved in initiation and progression of cancer [25,26]. In cervical cancer, increased expression of the nuclear (and thus active) forms of *RELA* (p65) and *NFKB1* (p50) was found to be associated with tumor progression and metastasis [27].

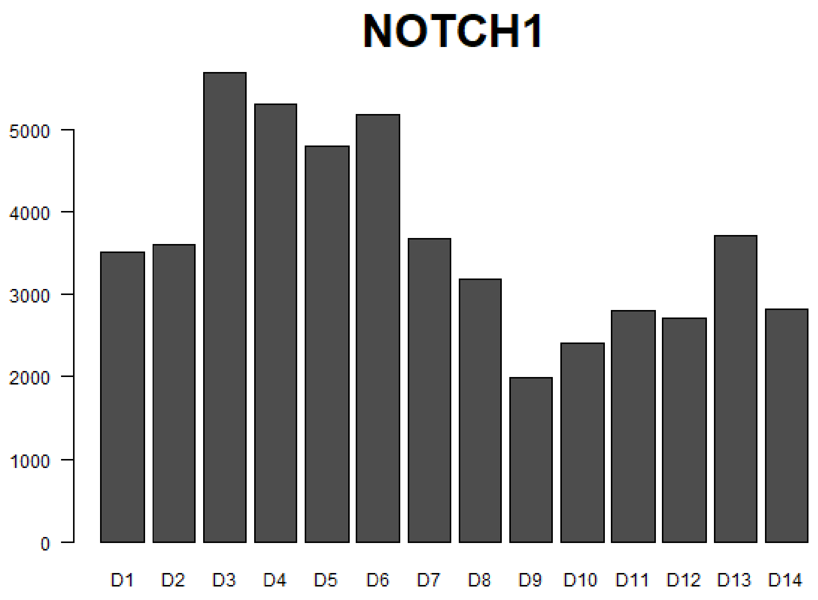

Figure S19: The gene *NOTCH1* is transiently over-expressed following release from cell cycle arrest and is associated with topic k2.

The gene *NOTCH1* was previously found to be over-expressed in cervical cancer with respect to normal cervical tissues [28,29].

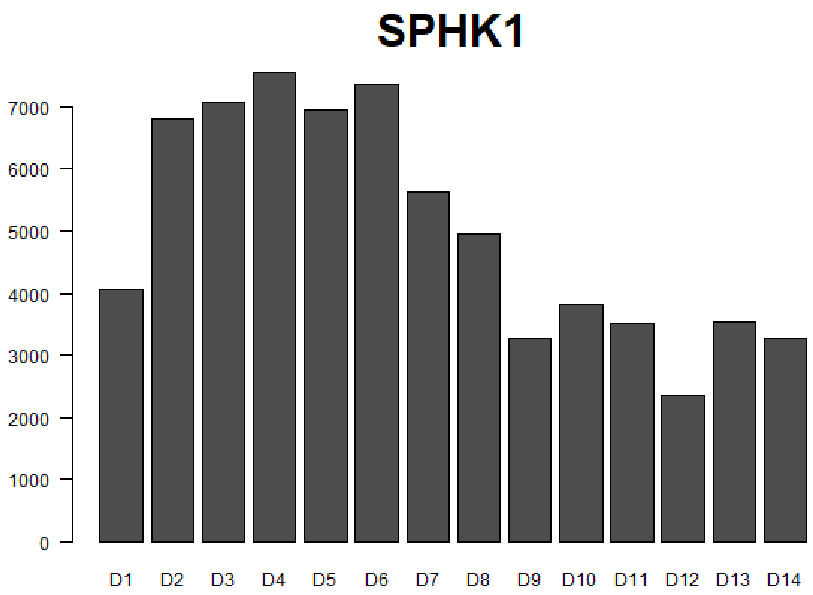

Figure S20: The gene *SPHK1* is transiently over-expressed following release from cell cycle arrest and is associated with topic k2.

The gene *SPHK1* is thought to promote inhibition of apoptosis and increased cell proliferation. This gene was previously found to be over-expressed in cervical cancer with respect to normal cervical tissue and to be associated with tumor stage, size, invasion, metastasis, and low survival [30].

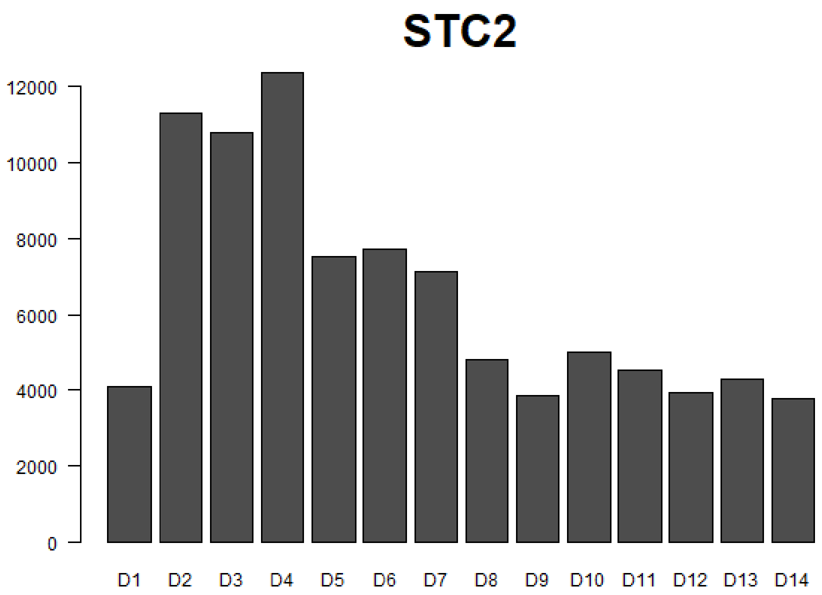

Figure S21: The gene *STC2* is transiently over-expressed following release from cell cycle arrest and is associated with topic k2.

The gene *STC2* was previously found to be significantly increased in cervical cancer tissues and cell lines compared to normal cervical tissues [31] and to promote proliferation in cervical cancer cell lines, including HeLa cells.

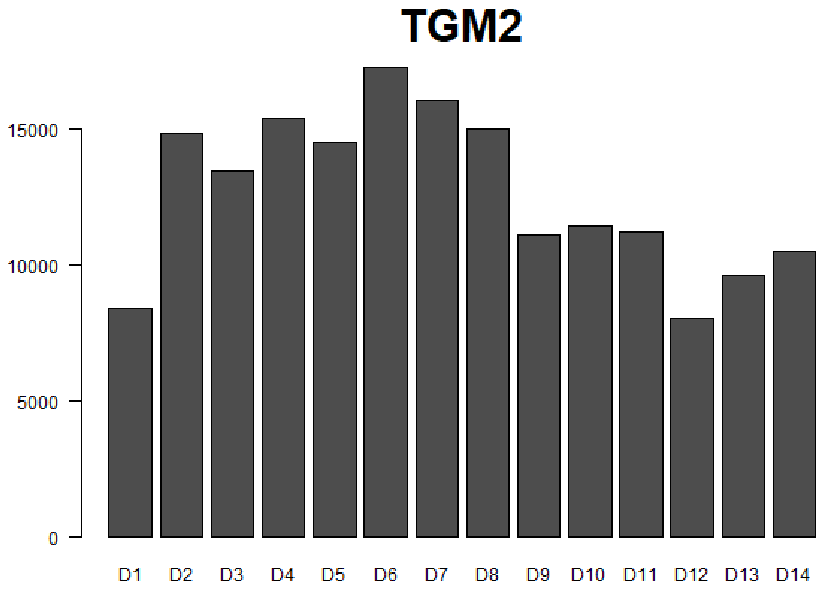

Figure S22: The gene *TGM2* is transiently over-expressed following release from cell cycle arrest and is associated with topic k2.

The gene *TGM2* was previously found to be over-expressed in cervical cancer relative to normal cervical samples [32].

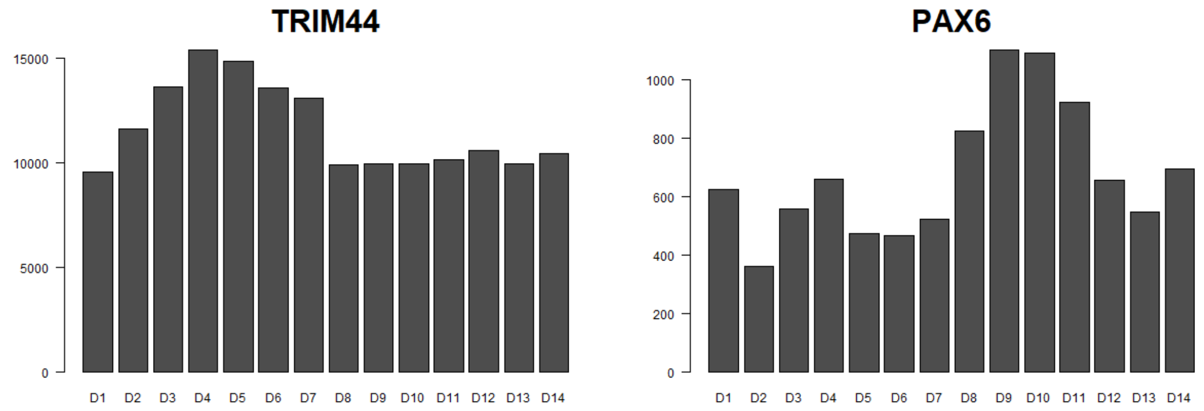

Figure S23: The gene *TRIM44* is transiently over-expressed following release from cell cycle arrest and is associated with topic k2.

The gene *TRIM44* was previously found to be significantly up-regulated in cervical cancer compared to normal cervical tissue, and also to be associated with increased tumor stage, grade, metastasis, and overall poor prognosis [33]. Interestingly, we also observed that *PAX6*, whose expression is thought to be inhibited by *TRIM44* [34], becomes transiently over-expressed later, after *TRIM44* over-expression has subsided (time points D8-D14).

.

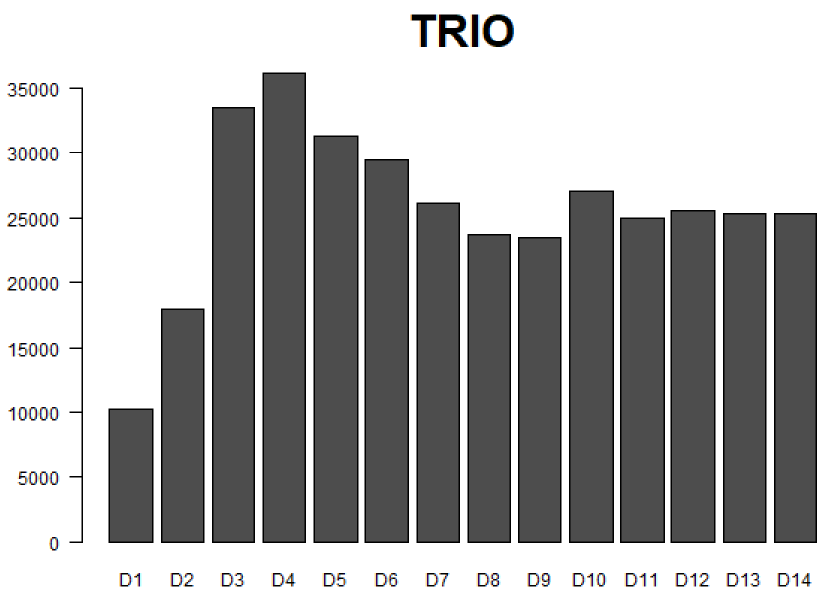

Figure S24: The gene *TRIO* is transiently over-expressed following release from cell cycle arrest and is associated with topic k2.

The gene *TRIO* was previously found to be over-expressed in cervical cancer with respect to adjacent normal tissues, and to be associated with cell migration [35] and metastasis [36]. Interestingly, we observed that during the first few hours after release from cell cycle arrest, the expression of this gene increases rapidly (time points D1-D4), 'overshoots', and then stabilizes during the second cell cycle (time points D8-D14).

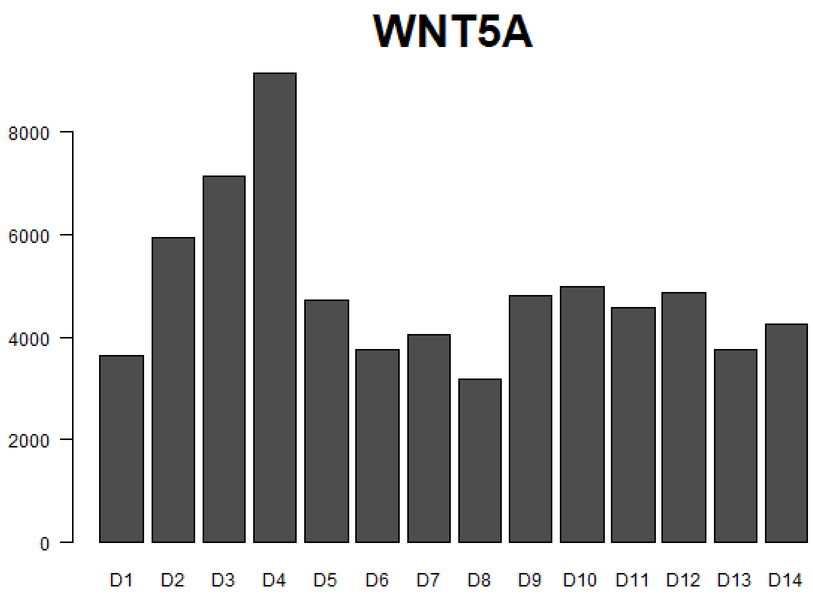

Figure S25: The gene *WNT5A* is transiently over-expressed following release from cell cycle arrest and is associated with topic k2.

The gene *WNT5A* was previously found to be overexpressed in cervical cancer compared to adjacent normal cervical tissues, and to be associated with tumor metastasis, recurrence, and shorter survival times [37].

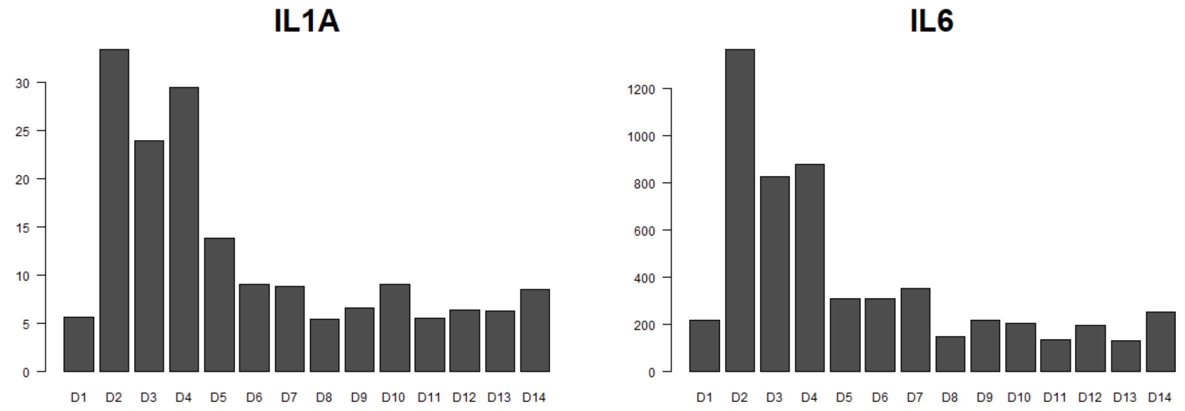

Figure S26: The genes *IL1A* and *IL6* are transiently over-expressed following release from cell cycle arrest and are associated with topic k2.

The gene *IL6* is an immediate-early response gene [7]. The genes *IL1A* and *IL6* were both previously found to be over-expressed in cervical cancer compared to adjacent non-tumor tissue, and to be associated with cervical cancer progression and shorter survival times [38].

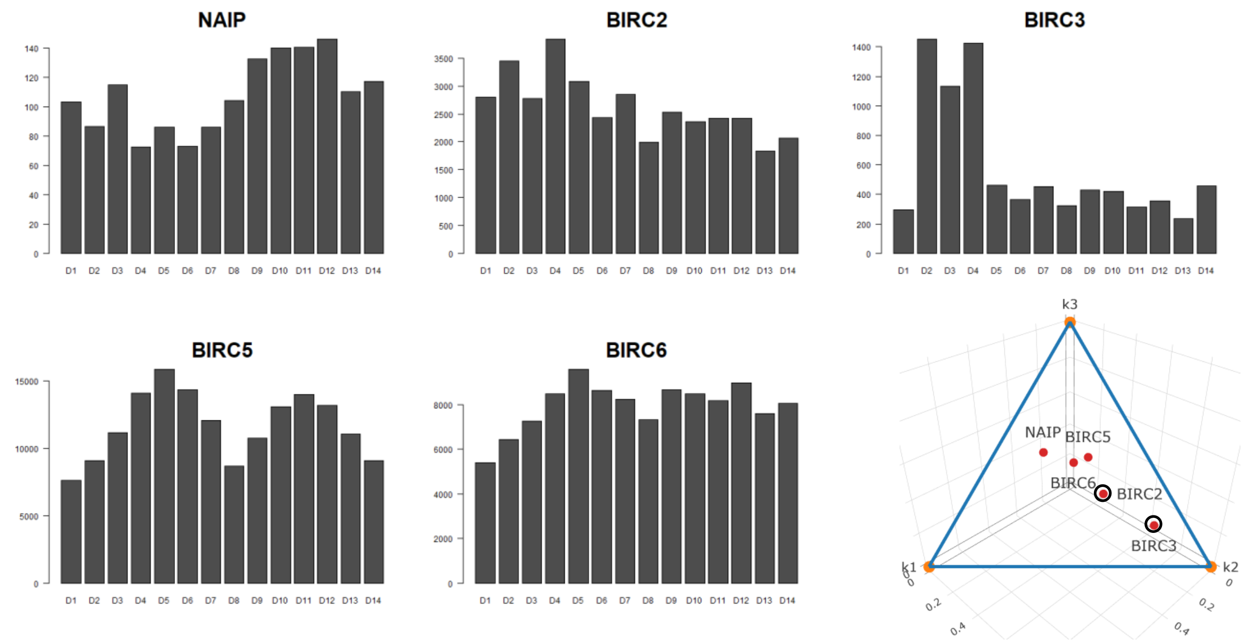

Figure S27: The genes *BIRC2* and *BIRC3* are transiently over-expressed following release from cell cycle arrest and are associated with topic k2.

The human BIR-containing protein family (BIRCs) is thought to contain two subgroups, each having structural and functional characteristics [39]. The first subgroup includes the genes *BIRC1* (NAIP), *BIRC2*, and *BIRC3*, which are presumed inhibitors of apoptosis, while the second group includes the genes *BIRC5* (Survivin) and *BIRC6* that are presumably regulators of the cell cycle and are required for mitotic chromosome segregation and cytokinesis. In the dataset that we studied we observed that the apoptosis inhibitors *BIRC2* and *BIRC3* are transiently over-expressed following release from cell cycle arrest, while the cell cycle regulator *BIRC5*, and to some extent also *BIRC6*, are periodically expressed. This can be seen also in the diagram of posterior probabilities (bottom right). Surprisingly, *BIRC1* (NAIP) seems to be transiently repressed following release from cell cycle arrest. These differences in expression and function may be attributed to structural differences, for example, *BIRC2* and *BIRC3* both contain a caspase-recruitment domain (CARD) whereas *BIRC1* (NAIP) has a nucleotide-binding loop instead [39].

REFERENCES

1. Winkles JA. Serum- and Polypeptide Growth Factor-Inducible Gene Expression in Mouse Fibroblasts. In: Moldave K, editor. Progress in Nucleic Acid Research and Molecular Biology. Academic Press; 1997. pp. 41–78. doi:10.1016/S0079-6603(08)60033-1

2. Bi L, Ma F, Tian R, Zhou Y, Lan W, Song Q, et al. AJUBA increases the cisplatin resistance through hippo pathway in cervical cancer. Gene. 2018;644: 148–154. doi:10.1016/j.gene.2017.11.017

3. Kalan S, Matveyenko A, Loayza D. LIM Protein Ajuba Participates in the Repression of the ATR-Mediated DNA Damage Response. Front Genet. 2013;4. doi:10.3389/fgene.2013.00095

4. Mehdi HK, Raju K, Sheela SR. Association of P16, Ki-67, and CD44 expression in high-grade squamous intraepithelial neoplasia and squamous cell carcinoma of the cervix. Journal of Cancer Research and Therapeutics. 2023;19: S260. doi:10.4103/jcrt.jcrt_43_21

5. Meyerson M, Harlow E. Identification of G1 kinase activity for cdk6, a novel cyclin D partner. Mol Cell Biol. 1994;14: 2077–2086.

6. Huang C, Chen Z, He Y, He Z, Ban Z, Zhu Y, et al. EphA2 promotes tumorigenicity of cervical cancer by up-regulating CDK6. Journal of Cellular and Molecular Medicine. 2021;25: 2967–2975. doi:10.1111/jcmm.16337

7. Tullai JW, Schaffer ME, Mullenbrock S, Sholder G, Kasif S, Cooper GM. Immediate-Early and Delayed Primary Response Genes Are Distinct in Function and Genomic Architecture. Journal of Biological Chemistry. 2007;282: 23981–23995. doi:10.1074/jbc.M702044200

8. Xie H, Zhao Y, Caramuta S, Larsson C, Lui W-O. miR-205 expression promotes cell proliferation and migration of human cervical cancer cells. PLoS One. 2012;7: e46990. doi:10.1371/journal.pone.0046990

9. Wong Y-F, Cheung T-H, Tsao GSW, Lo KWK, Yim S-F, Wang VW, et al. Genome-wide gene expression profiling of cervical cancer in Hong Kong women by oligonucleotide microarray. Int J Cancer. 2006;118: 2461–2469. doi:10.1002/ijc.21660

10. Fernandez-Avila L, Castro-Amaya AM, Molina-Pineda A, Hernández-Gutiérrez R, Jave-Suarez LF, Aguilar-Lemarroy A. The Value of CXCL1, CXCL2, CXCL3, and CXCL8 as Potential Prognosis Markers in Cervical Cancer: Evidence of E6/E7 from HPV16 and 18 in Chemokines Regulation. Biomedicines. 2023;11: 2655. doi:10.3390/biomedicines11102655

11. Kim JW, Kim YT, Kim DK, Song CH, Lee JW. Expression of Epidermal Growth Factor Receptor in Carcinoma of the Cervix. Gynecologic Oncology. 1996;60: 283–287. doi:10.1006/gyno.1996.0039

12. Shen L, Shui Y, Wang X, Sheng L, Yang Z, Xue D, et al. EGFR and HER2 expression in primary cervical cancers and corresponding lymph node metastases: Implications for targeted radiotherapy. BMC Cancer. 2008;8: 232. doi:10.1186/1471-2407-8-232

13. Zhang L, Chen Q, Hu J, Chen Y, Liu C, Xu C. Expression of HIF-2α and VEGF in Cervical Squamous Cell Carcinoma and Its Clinical Significance. Biomed Res Int. 2016;2016: 5631935. doi:10.1155/2016/5631935

14. Fujimoto J, Ichigo S, Hori M, Hirose R, Sakaguchi H, Tamaya T. Expression of basic fibroblast growth factor and its mRNA in advanced uterine cervical cancers. Cancer Letters. 1997;111: 21–26. doi:10.1016/S0304-3835(96)04485-0

15. Casalino L, Talotta F, Cimmino A, Verde P. The Fra-1/AP-1 Oncoprotein: From the "Undruggable" Transcription Factor to Therapeutic Targeting. Cancers. 2022;14: 1480. doi:10.3390/cancers14061480

16. Milde-Langosch K. The Fos family of transcription factors and their role in tumourigenesis. European Journal of Cancer. 2005;41: 2449–2461. doi:10.1016/j.ejca.2005.08.008

17. O'Donnell A, Odrowaz Z, Sharrocks AD. Immediate-early gene activation by the MAPK pathways: what do and don't we know? Biochem Soc Trans. 2012;40: 58–66. doi:10.1042/BST20110636

18. Yee GPC, De Souza PL, Khachigian LM. Reducing invasion potential of cervical cancer cells via targeted knockdown of c-Jun. JCO. 2013;31: e22005–e22005. doi:10.1200/jco.2013.31.15_suppl.e22005

19. Prusty BK, Das BC. Constitutive activation of transcription factor AP-1 in cervical cancer and suppression of human papillomavirus (HPV) transcription and AP-1 activity in HeLa cells by curcumin. International Journal of Cancer. 2005;113: 951–960. doi:10.1002/ijc.20668

20. Liao H, Zhang L, Lu S, Li W, Dong W. KIFC3 Promotes Proliferation, Migration, and Invasion in Colorectal Cancer via PI3K/AKT/mTOR Signaling Pathway. Front Genet. 2022;13: 848926. doi:10.3389/fgene.2022.848926

21. Halle MK, Sødal M, Forsse D, Engerud H, Woie K, Lura NG, et al. A 10-gene prognostic signature points to LIMCH1 and HLA-DQB1 as important players in aggressive cervical cancer disease. Br J Cancer. 2021;124: 1690–1698. doi:10.1038/s41416-021-01305-0

22. Wang L, Zhong Y, Yang B, Zhu Y, Zhu X, Xia Z, et al. LINC00958 facilitates cervical cancer cell proliferation and metastasis by sponging miR-625-5p to upregulate LRRC8E expression. Journal of Cellular Biochemistry. 2020;121: 2500–2509. doi:10.1002/jcb.29472

23. de Cárcer G, Manning G, Malumbres M. From Plk1 to Plk5. Cell Cycle. 2011;10: 2255–2262. doi:10.4161/cc.10.14.16494

24. Weerdt BCM van de, Medema RH. Polo-Like Kinases: A Team in Control of the Division. Cell Cycle. 2006;5: 853–864. doi:10.4161/cc.5.8.2692

25. Costa RMGD, Bastos MMSM, Medeiros R, Oliveira PA. The NFκB Signaling Pathway in Papillomavirus-induced Lesions: Friend or Foe? Anticancer Research. 2016;36: 2073–2083.

26. Tilborghs S, Corthouts J, Verhoeven Y, Arias D, Rolfo C, Trinh XB, et al. The role of Nuclear Factor-kappa B signaling in human cervical cancer. Critical Reviews in Oncology/Hematology. 2017;120: 141–150. doi:10.1016/j.critrevonc.2017.11.001

27. Li J, Jia H, Xie L, Wang X, Wang X, He H, et al. Association of Constitutive Nuclear Factor-κB Activation With Aggressive Aspects and Poor Prognosis in Cervical Cancer: International Journal of Gynecological Cancer. 2009;19: 1421–1426. doi:10.1111/IGC.0b013e3181b70445

28. Sun Y, Zhang R, Zhou S, Ji Y. Overexpression of Notch1 is associated with the progression of cervical cancer Corrigendum in /10.3892/ol.2020.12395. Oncology Letters. 2015;9: 2750–2756. doi:10.3892/ol.2015.3143

29. Zagouras P, Stifani S, Blaumueller CM, Carcangiu ML, Artavanis-Tsakonas S. Alterations in Notch signaling in neoplastic lesions of the human cervix. Proc Natl Acad Sci U S A. 1995;92: 6414–6418.

30. Kim H-S, Yoon G, Ryu J-Y, Cho Y-J, Choi J-J, Lee Y-Y, et al. Sphingosine kinase 1 is a reliable prognostic factor and a novel therapeutic target for uterine cervical cancer. Oncotarget. 2015;6: 26746–26756.

31. Wang Y, Gao Y, Cheng H, Yang G, Tan W. Stanniocalcin 2 promotes cell proliferation and cisplatin resistance in cervical cancer. Biochemical and Biophysical Research Communications. 2015;466: 362–368. doi:10.1016/j.bbrc.2015.09.029

32. Caffarel MM, Chattopadhyay A, Araujo AM, Bauer J, Scarpini CG, Coleman N. Tissue transglutaminase mediates the pro-malignant effects of oncostatin M receptor over-expression in cervical squamous cell carcinoma. J Pathol. 2013;231: 168–179. doi:10.1002/path.4222

33. Liu S, Meng F, Ding J, Ji H, Lin M, Zhu J, et al. High TRIM44 expression as a valuable biomarker for diagnosis and prognosis in cervical cancer. Biosci Rep. 2019;39: BSR20181639. doi:10.1042/BSR20181639

34. Zhang X, Qin G, Chen G, Li T, Gao L, Huang L, et al. Variants in TRIM44 Cause Aniridia by Impairing PAX6 Expression. Human Mutation. 2015;36: 1164–1167. doi:10.1002/humu.22907

35. van Rijssel J, van Buul JD. The many faces of the guanine-nucleotide exchange factor trio. Cell Adh Migr. 2012;6: 482–487. doi:10.4161/cam.21418

36. Hou C, Zhuang Z, Deng X, Xu Y, Zhang P, Zhu L. Knockdown of Trio by CRISPR/Cas9 suppresses migration and invasion of cervical cancer cells. Oncology Reports. 2018;39: 795–801. doi:10.3892/or.2017.6117

37. Lin L, Liu Y, Zhao W, Sun B, Chen Q. Wnt5A expression is associated with the tumor metastasis and clinical survival in cervical cancer. Int J Clin Exp Pathol. 2014;7: 6072–6078.

38. Song Z, Lin Y, Ye X, Feng C, Lu Y, Yang G, et al. Expression of IL-1α and IL-6 is Associated with Progression and Prognosis of Human Cervical Cancer. Med Sci Monit. 2016;22: 4475–4481. doi:10.12659/MSM.898569

39. Silke J, Vaux DL. Two kinds of BIR-containing protein - inhibitors of apoptosis, or required for mitosis. Journal of Cell Science. 2001;114: 1821–1827. doi:10.1242/jcs.114.10.1821
